# Ex vivo evolution of human antibodies by CRISPR-X: from a naive B cell repertoire to affinity matured antibodies

**DOI:** 10.1186/s12896-019-0504-z

**Published:** 2019-02-18

**Authors:** Marie-Claire Devilder, Melinda Moyon, Laetitia Gautreau-Rolland, Benjamin Navet, Jeanne Perroteau, Florent Delbos, Marie-Claude Gesnel, Richard Breathnach, Xavier Saulquin

**Affiliations:** 1 CRCINA, INSERM, CNRS, Université d’Angers, Université de Nantes, Nantes, France; 2 LabEx IGO “Immunotherapy, Graft, Oncology”, Nantes, France; 3 Centre Hospitalier Universitaire Hôtel-Dieu, Nantes, France; 4 HLA Laboratory, EFS Centre Pays de la Loire, Nantes, France

**Keywords:** Human antibodies, SHM, CRISPR, CRISPR-X, AID, Tetramers, HLA, Cytofluorimetry

## Background

Somatic hypermutation promotes affinity maturation of antibodies by targeting the cytidine deaminase AID to antibody genes, followed by antigen-based selection of matured antibodies. Given the importance of antibodies in medicine and research, developing approaches to reproduce this natural phenomenon in cell culture is of some interest.

## Results

We use here the CRISPR-Cas 9 based *CRISPR-X* approach to target AID to antibody genes carried by expression vectors in HEK 293 cells. This directed mutagenesis approach, combined with a highly sensitive antigen-associated magnetic enrichment process, allowed rapid progressive evolution of a human antibody against the Human Leucocyte Antigen A*02:01 allele. Starting from a low affinity monoclonal antibody expressed on Ag-specific naïve blood circulating B cells, we obtained in approximately 6 weeks antibodies with a two log increase in affinity and which retained their specificity.

## Conclusion

Our strategy for in vitro affinity maturation of antibodies is applicable to virtually any antigen. It not only allows us to tap into the vast naive B cell repertoire but could also be useful when dealing with antigens that only elicit low affinity antibodies after immunization.

## Background

The human B cell repertoire constitutes a source of antibodies capable of recognizing virtually any antigen (Ag). This is the result of a complex B lymphocyte maturation process. Newly produced B cells express B cell receptors (BCRs) generated by random somatic recombination of V (Variable), D (Diversity) and J (Junction) gene segments and which generally have a low affinity for their cognate Ag [1]. After exposure to an Ag, naïve B cells with Ag-specific BCRs undergo somatic hypermutation (SHM) catalyzed by the enzyme Activation induced cytidine deaminase (AID) [2–4]. This enzyme is targeted to the Ig-loci in B cells and deaminates cytosines, thus provoking point mutations, insertions and deletions in the variable domains of both the heavy and light chains. This process ultimately leads to antibody diversification and is followed by the selection of a matured B cell repertoire with higher affinity and specificity for the Ag. This allows the overall diversity of the BCR / antibody molecules to reach theoretically about 10^13^ different receptors in humans [5]. The repertoire thus constitutes an almost unlimited resource of antibodies.

For several decades, monoclonal antibodies (mAb) have been crucial tools in the treatment of diseases such as autoimmune diseases and cancer, or for the control of graft rejection. It is important to generate fully human mAbs because they have a lower risk of immune response induction in humans than the mouse, chimeric or humanized mAbs generally used hitherto. Various methods have been developed for isolating antibodies directly from a natural repertoire of human B lymphocytes. In general, they derive from two main approaches. The first of these is the high-throughput screening of mAb produced by B cell cultures or plasma cells [6, 7]. This is a very effective method for obtaining mAb against Ag to which an individual is exposed naturally or by vaccination. However, many Ag of therapeutic interest are not encountered sufficiently frequently naturally, or exploitable in vaccine strategies in humans, to profit from this type of methodology. The second technique consists in isolating single Ag-specific B cells using fluorescent-tagged Ag, followed by cloning of their immunoglobulin genes and expression of recombinant antibodies in a cell line. This technique allows interrogation of both the immune/matured B cell repertoire and the naïve/germline repertoire of an individual with respect to any Ag available in purified form [8–10]. There is a limitation to the interrogation of a naive B cell repertoire however: the generally limited affinity of the corresponding recombinant antibodies, requiring identification of mutations that enhance affinity while maintaining specificity.

Antibody optimization currently relies heavily on the use of libraries generated by mutagenesis of antibody chains using error-prone PCR or degenerate primers. Libraries are screened using techniques such as ribosome, phage, yeast or mammalian display [11]. Co-expression of AID and antibody or non-antibody genes in various mammalian cell lines has also been used to initiate a mutagenic process mimicking SHM [12–20]. This approach circumvents the need to construct mutant libraries, but does not allow targeting of the AID enzyme to sequences encoding the antibody. In B cells, AID is targeted to the immunoglobulin locus by complex mechanisms not yet fully elucidated [21].

We wanted to develop a simple strategy for AID-targeting to antibody sequences in non-B cells to obtain mutated antibodies with increased affinity. Various CRISPR Cas9-based approaches using guide RNAs to target base editors such as APOBEC or AID fused to dead Cas9 (dCas9) to specific DNA sequences have been described recently [22, 23]. These approaches generally lead to mutations limited to a small part of the sequences corresponding to the guide RNA binding site. A variant approach (CRISPR-X) uses a complex containing dCas9 and a guide RNA containing bacteriophage MS2 coat protein binding sites to recruit a coat-AID fusion to DNA [24]. This leads to more extensive mutagenesis covering a window of approximately 100 bp around the guide RNA binding site.

In this work, we present a CRISPR-X based strategy for targeted *in cellulo* affinity maturation of low affinity human mAbs. We apply it to a low affinity mAb named A2Ab against HLA-A*02:01 which shows some crossreactivity against other HLA-A alleles. A2Ab was isolated from circulating B cells of a naïve individual using a procedure recently developed by our group [8, 10]. We used CRISPR-X with multiplexed guide RNAs to target AID to the VDJ segment encoding the A2Ab heavy chain variable domain in HEK 293 cells co-expressing the light chain. This directed-mutagenesis approach, combined with mammalian surface expression display and a very sensitive Ag-associated magnetic enrichment process, allowed us to identify mAbs with increased affinity and a sharpening of their specificity for HLA-A*02:01. Overall we describe a novel procedure for generation of high-affinity/optimized human mAbs that is applicable to both naïve and mature circulating human B cells, raising the possibility of generation of private antibodies from a particular individual.

## Methods

### Donors

Human peripheral blood samples were obtained from anonymous adult donors after informed consent in accordance with the local ethics committee (Etablissement Français du Sang, EFS, Nantes, procedure PLER NTS-2016-08).

### Cell lines and culture conditions

Human embryonic kidney 293A cells were obtained from Thermo Fisher Scientific, San Jose, CA, USA (R70507). Cells were grown as adherent monolayers in DMEM (4.5 g/l glucose) supplemented with 10% FBS, 1% Glutamax (Gibco) and 1% penicillin (10,000 U/ml)/streptomycin (10,000 U/ml) (a mixture from Gibco). The BLCL cell lines HEN (HLA-A*02:01/ HLA-A*0101), B721.221 and stably transfected HLA-A2 B721.221 (B721.221 A2) were grown in suspension in RPMI medium supplemented with 10% FBS, 1% Glutamax (Gibco) and 1% penicillin (10,000 U/ml)/streptomycin (10,000 U/ml) (a mixture from Gibco).

### Plasmid constructions

Plasmids for mutagenesis were obtained from Addgene: pGH335_MS2-AID*Δ-Hygro (catalogue n° 85.406), pX330S-2 to 7 from the Multiplex CRISPR/Cas9 Assembly System kit (n° 1.000.000.055) and pX330A_dCas9-1 × 7 from the multiplex CRISPR dCas9/Fok-dCas9 Accessory pack (n° 1.000.000.062). The sgRNA scaffolds in the seven latter plasmids were replaced by the sgRNA_2MS2 scaffold from pGH224_sgRNA_2xMS2_Puro (Addgene n° 85.413) and guide sequences then introduced into their BbsI sites before Golden Gate assembly. SgRNA design was performed online using Sequence Scan for CRISPR software (http://crispr.dfci.harvard.edu/SSC/). Final plasmids for mutagenesis thus obtained contain expression cassettes for dCas9 and seven sgRNAs. For production of antibodies, VH and VL regions from human antibodies were subcloned respectively in an IgG-Abvec expression vector (FJ475055) and an Iglambda –AbVec expression vector (FJ51647) as previously described [8]. For mammalian display of antibodies as IgG1, VH and VL regions were subcloned into home-made expression vectors derived from the OriP/EBNA1 based episomal vector pCEP4. The VH and VL expression vectors contain a hygromycin B or Zeocin resistance marker respectively, and a transmembrane region encoding sequence exists in the C gamma constant region sequence.

### IgG1 mammalian cell display

Heavy and light chain expression vectors were co-transfected into the 293A cell line at a 1:1 ratio using JetPEI (PolyplusTransfection, Cat. 101–10 N) and cultured for 48 h. Selection of doubly transfected cells was performed using Hygromycin B and Zeocin. Antibody surface expression on the selected cells was confirmed by flow cytometry analysis after staining with a PE-labeled goat-anti-human IgG Fc (Jackson ImmunoResearch).

### Peptide MHC tetramer

The HLA-A*02:01–restricted peptides Pp65_495_ (human CMV [HCMV], NLVPMVATV) and MelA27 (melanoma Ag, ELAGIGILTV) and the HLA-B*0702-restricted UV-sensitive peptide (AARGJTLAM; where J is 3-amino-3-(2-nitro)phenyl-propionic acid) were purchased from GL Biochem (Shanghaï, China). Soluble peptide MHC monomers used in this study carried a mutation in the α3 domain (A245V), that reduces CD8 binding to MHC class I. Biotinylated HLA-A*02:01/MelA_27_ (HLA-A2/MelA), HLA-A*02:01/Pp65_495_ (HLA-A2/Pp65), HLA-B*0702/UV sensitive peptide (HLA-B7/pUV) monomers were tetramerized with allophycocyanin (APC)-labeled premium grade streptavidins (Molecular Probes, Thermo Fischer Scientific, ref. S32362) at a molar ratio of 4:1. When applicable, the avidity of the tetramer for its specific antibody was decreased by mixing specific (ie peptide HLA-A2) and unspecific (ie peptide UV-sensitive HLA-B7) biotinylated monomers before tetramerization with APC-labeled streptavidins at different molar ratios.

### Ag-specific B cell sorting from PBMC

B cell isolation was performed as previously described [8, 10]. Briefly, PBMCs were obtained by Ficoll density gradient centrifugation and incubated with PE-, APC and BV421-conjugated tetramers (10 μg/mL in PBS plus 2% FBS, for 30 min at room temperature). The tetramer-stained cells were enriched using anti-PE and-APC Ab-coated paramagnetic beads and then stained with anti-CD19-PerCpCy5.5 (BD Biosciences) mAbs. Stained samples were collected on an ARIA Cell Sorter Cytometer (BD Biosciences) and single CD19^+^ CD3^−^ PE^+^ APC^+^ BV421^−^ tetramer cells were collected in individual PCR tubes.

### Flow cytometry analysis

The specificity and avidity of IgG expressing HEK 293 cells was analysed by flow cytometry. Cells were first stained in PBS containing 0.5% BSA with Ag tetramers for 30 min at room temperature. Anti-PE human IgG was then added at a 1/500 dilution for 15 min on ice without prior washing. The binding of mutant antibodies was evaluted on 150,000 BLCL cells. Cells were incubated with various concentrations of large-scale purified mAbs diluted in 25 ml of PBS containing 0.5% BSA for 30 min at room temperature. Anti-PE goat anti-human IgG was then added at a 1/500 dilution for 15 min on ice without prior washing.

### Mutagenesis

4 × 10^6^ anti HLA-A2 IgG-expressing cells were seeded the day before transfection in a 175 cm flask. For each round of mutation, cells were transiently transfected using JET-PRIME (PolyplusTransfection, Cat. 101–10 N) with pGH335_MS2-AID*Δ-Hygro together with two other plasmids allowing expression of a total of 9 different sgRNAs along with dCas9 at a ration 1: 1: 1.

### Affinity-based cell selection and immunomagnetic enrichment

After a round of mutagenesis, transfected cells were expanded until confluency over a week. For selection, 10-20 × 10^6^ cells were washed, resuspended in 0.2 mL of PBS containing 2% BSA and the antigen (i.e. APC HLA-A2 tetramers or mixed APC HLA-A2/HLA-B7 tetramers) and incubated for 30 min at room temperature. The tetramer-stained cells were then positively enriched using anti APC Ab-coated immunomagnetic beads and columns as previously described [8]. The resulting enriched fraction was stained with an anti human IgG-PE. IgG PE+ and tetramer APC+ cells were collected on an ARIA cell sorter. The adopted strategy for evolution of mAb A2Ab was as follows: 1) three rounds of mutagenesis; 2) magnetic enrichment with 3A2/1B7 tetramer; 3) FACS sorting of positive cells. Positively selected and sorted mutated HEK 293 underwent two new rounds of mutation using the same sgRNAs before selection with the 1A2/3B7 tetramer.

### Antibody production

Antibody production was performed as previously described [8]. Briefly, 293A cell lines were transiently transfected with VH and VL expression vectors and cultured for 5 days in serum free medium in 175 cm2 flasks. Recombinant antibodies produced were purified from cell supernatant by Fast Protein Liquid Chromatography (FPLC) using a protein A column, and their concentration determined by absorbance measurement at 280 nm.

### Elisa

96-well ELISA plates (Maxisorp, Nunc) were coated with HLA-A2 monomers (overnight at 4 °C, final concentration 2 μg/mL in a coating buffer 1X (Affymetrix)), saturated with a 10% FBS DMEM blocking buffer (Thermo Fischer Scientific) for 2 h at 37 °C and (iii) incubated with serial dilutions of purified mAbs for 2 h at room temperature. Binding of mAbs was detected with an anti-human IgG-HRP Ab (BD Bioscience, 1 μg/mL, 1 h) and addition of a chromogenic substrate for 20 min at room temperature (Maxisorp, Nunc).

### Anti–HLA antibody testing (Luminex)

A Single Antigen Flow Bead assay (LabScreen single-antigen LS1A04, One Lambda, Inc., Canoga Park, CA), was used to detect anti-HLA antibodies in donors and test the specificity of antibodies against 97 MHC-class I alleles. Analysis was performed with a Luminex 100 analyser (Luminex, Austin, TX) after removal of the background as previously described [10].

### Surface Plasmon resonance

Surface Plasmon Resonance (SPR) experiments were performed on a Biacore 3000 apparatus (GE Healthcare Life Sciences, Uppsala, Sweden) on CM5 chips (GE Healthcare) as previously described [10]. Briefly, mAbs were immobilized at 10 μg/mL The sensor chip surface was then deactivated and various dilutions of HLA-A*02:01 peptide monomers were injected for 180 s at 40 μL/min.

### Bioinformatics analysis

Amplicon preparation: total RNA was purified from 5 × 10^6^ HEK 293 cells and 1 μg of total RNA was reverse transcribed using Superscript reverse transcriptase III (ThermoFisher). cDNA was subsequently amplified using Q5 DNA polymerase and primers targeting VH sequences. Sense and antisense primers include target sequences suitable for Nextera indexage. Barcodes were further introduced by PCR with indexed nextera and the amplicons were sequenced at the IRIC’s Genomics Core Facility at Montreal. Paired-end MiSeq technology (Miseq Reagent Nano kit v2 (500 cycles) from Illumina, Inc. San Diego, CA, USA) was used, with a 2 × 250 bp setup.

### Pretreatment and sequence clustering

For each chip generated, approximately one million reads were obtained for all the samples. The quality and length distribution of the reads were checked using the FASTQ tool (v0.11.7). After that, for each sample, the paired-end sequences were assembled using the PEAR software (v0.9.6) while keeping only the sequences whose Phred score was greater than 33 and whose overlap was at least 10 nucleotides. Then 30,000 sequences were randomly selected to normalize samples. Next, for each sample, full length VH sequences were grouped according to their identity and counted and clusters were formed as described in the text. Mutations observed in the mock control (gRNA only) experiment were then eliminated in order to distinguish site-directed mutations from RT-PCR or sequence errors. Only clusters representing more than 0.1% of the total number of sequences were retained.

### Alignment and mutation analysis

For each sample, the generated clusters were annotated by aligning each sequence cluster against the reference sequence using Biostring library (v2.48.0) in a custom R script, to generate a counting table. The generated data were filtered by subtracting the mutations detected in the mock sample. A position matrix was then generated to create a Weblogo using the ggseqlogo library (v0.1). The data processing was performed using a custom R script.

## Results

### Isolation of a low affinity human antibody against HLA-A*02:01

**Fig. 1:**
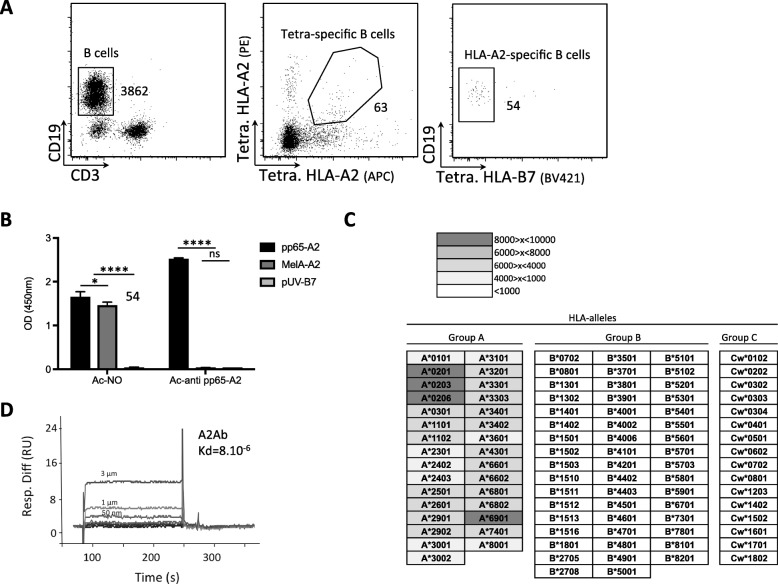
Isolation and characterization of human mAb A2Ab. **a** Sorting strategy used to isolate HLA-A2-specific B lymphocytes from donor NO. Cells with the following phenotypic characteristics: CD3-, CD19+ (left panel), both PE and APC labeled HLA-A2 tetramers+ (middle panel), HLA-B7 tetramer BV421- (right panel) were isolated and used to produce recombinant antibodies. **b** A2Ab Ab in Fig. 1b and a control anti- pp65-HLA-A*02:01 human mAb (Ac-anti pp65-A2) were tested by ELISA against the following peptide-MHC recombinant monomers: pp65-HLA-A*02:01 (pp65-A2), MelA-HLA-A*02:01 (MelA-A2) and pUV-HLA-B*0701 (pUV-B7). Statistical significance was determined using a two-way ANOVA test followed by a Tukey’s multiple comparison post-test (*n* = 3, bars indicate standard deviations) (****: *p* < 0.0001; *:*p* = 0,0143; ns: not significant). **c**) The specificity of A2Ab was assessed in a Luminex single antigen bead assay. Results are shown in terms of interval MFI. Positivity threshold was set at 1000. **d** The affinity of A2Ab was measured by surface plasmon resonance by flowing various concentrations of pp65-A2 complex over CM5 chip-bound A2Ab

A human HLA-A*02:01 molecule (hereafter referred to as HLA-A2) was selected as a target for antibody discovery and maturation as it is easy to obtain blood samples from donors not previously immunized against this MHC allele. In addition, various recombinant HLA molecules were readily available in our laboratory. PBMCs from three HLA-A2-negative donors with negative serology for HLA-A2 circulating antibodies (Additional file 1: Table S1) were tested for the presence of blood circulating B cells specific for HLA-A2. This was done by flow cytometry sorting of B cells that bound HLA-A2 tetramers labeled with two different fluorochromes but did not bind HLA-B7 tetramers, using a technique described previously [8, 10]. B lymphocytes stained specifically by HLA-A2 tetramers could be identified in PBMC from all three donors (see Fig. 1a for an example) and were isolated as single cells. We attempted RT-PCR amplification of sequences coding for the variable regions of the heavy and light chains of four B lymphocytes isolated from one donor (NO) using a recently published protocol [8, 10]. A pair of heavy and light chain V region coding sequences was obtained for one of the four cells. After cloning these gene segments into eukaryotic expression vectors in phase with human heavy and light chain constant domains, the corresponding antibody (A2Ab) was successfully produced in the supernatant of transfected HEK cells and tested for its specificity**.** A2Ab recognizes HLA-A2 but not HLA-B7 in ELISA tests and this recognition does not depend on the peptide loaded into the HLA pocket (Fig. 1b). A single HLA antigen flow bead assay analysis confirmed that A2Ab can recognize HLA-A*02:01, but also showed that A2Ab recognizes closely related alleles belonging to the HLA-A*02 supertype (HLA-A*02:03, A*02:06 and A*69:01) and weakly cross-reacts with other MHC A alleles. However, B or C alleles are not recognized (data not shown, results summarized in Fig. 1c). Finally, the affinity of A2Ab for the pp65/HLA-A2 complex was determined by surface plasmon resonance (SPR) to be in the low micromolar range (Kd = 8.10^− 6^, Fig. 1d). This is consistent with the HLA-A2-specific B cells being isolated from a naive/non-immune blood circulating B cell repertoire. The full nucleotide sequences of the heavy and light chains are provided in Additional file 1: Table S2.

### CRISPR-X targeted mutagenesis of A2Ab and screening for higher avidity antibodies

**Fig. 2:**
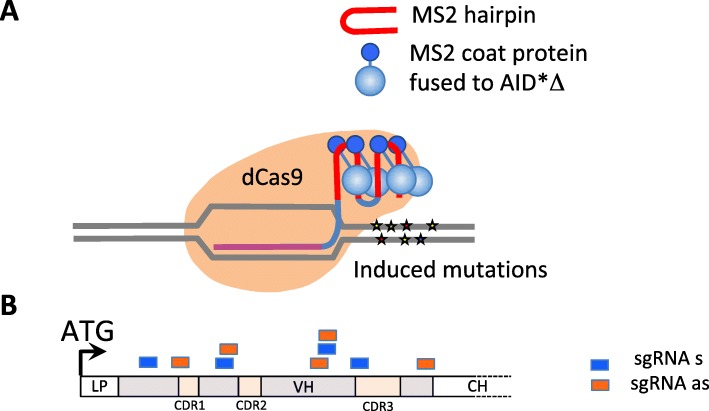
Schematic illustration of CRISPR-X. **a** dCas9 associated with a sgRNA containing MS2 hairpins recruits AID*Δ fused to MS2 coat protein leading to localized mutations (stars). Mutations can be induced in the sgRNA binding site or upstream or downstream from it, though only downstream mutations are illustrated here. **b** Binding sites for the nine sgRNAs used on the A2Ab heavy chain variable domain coding sequence are shown. Blue and orange colors indicate complementarity to non-coding and coding strands respectively

**Fig. 3:**
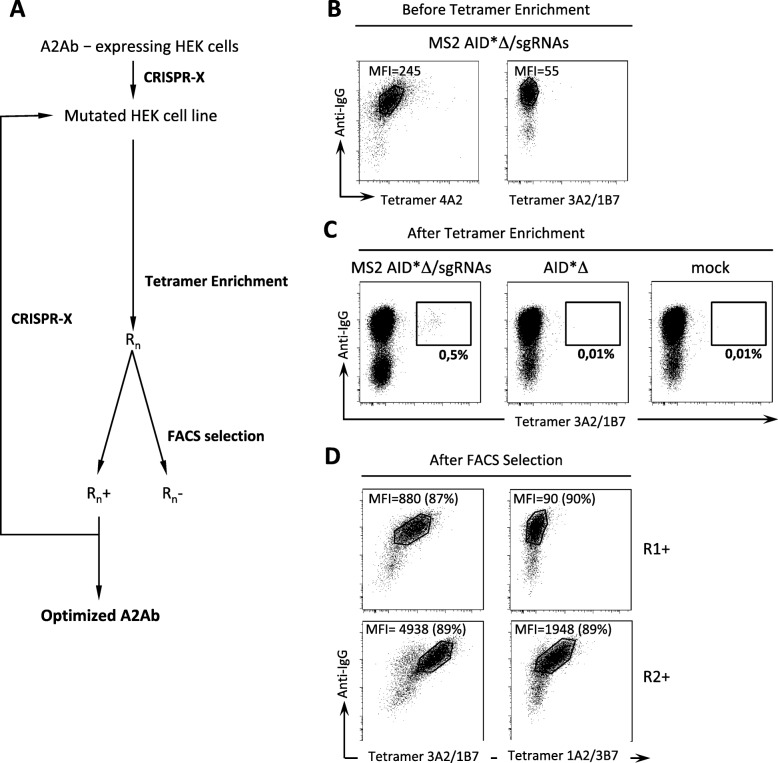
Generation and selection of HEK 293 cells expressing affinity-matured antibodies. **a** Overall strategy for antibody affinity maturation. HEK 293 cells expressing the initial Ab are subjected to CRISPR-X mutagenesis. Cells expressing variant antibodies of higher avidity are enriched using Stringent Tetramer-Associated Magnetic Enrichment (S-TAME) and expanded in vitro (R for “ enriched population”, subscript n for round of mutation/selection). Enriched cells are separated by FACS into tetramer positive-staining (R+) and tetramer negative-staining (R-) populations. Multiple rounds of mutation/selection can be performed successively as indicated. **b** Staining of A2Ab-expressing HEK 293 cells with 4A2-tetramers or 3A2/1B7 tetramers as marked after 3 successive transfections for CRISPR-X mutagenesis. Results shown are before the S-TAME step. **c** Staining of cells with tetramer 3A2/1B7 after S-TAME. Results are shown for cells transfected with dCas9, sgRNAs and MS2 AID*Δ (R1 cells, left panel), AID*Δ alone (middle panel) and sgRNA alone (right panel). **d** Cells from the R1 population staining positive with the 3A2/1B7 tetramer were isolated by FACS (R1+ cells). Staining of these cells with tetramers 3A2/1B7 (upper left panel) and 1A2/3B7 (upper right panel) is shown. R1+ cells were subjected to a second round of mutagenesis, S-TAME and FACS selection to generate R2+ cells. Staining of R2+ cells with tetramers 3A2/1B7 (lower left panel) and 1A2/3B7 lower right panel) is shown. The number of cells within marked gates is shown between brackets as a percentage of the total cells analysed

We used the CRISPR-X approach [24] (Fig. 2a) to mutate the A2Ab sequence. Our overall procedure using iterative mutation and selection is summarized in Fig. 3a. HEK 293 cells were engineered to express cell surface A2Ab by stable transfection of episomal vectors expressing its heavy and light chains (HC and LC, respectively). For induction of mutations, these cells were then transiently transfected with a plasmid coding for AID*Δ fused to MS2 coat protein, and plasmids coding for dCas9 and nine different sgRNAs (Additional file 1: Table S3) spanning the sequence coding for the A2Ab HC variable domain (Fig. 2b). AID*Δ is an AID mutant with increased SHM activity whose Nuclear Export Signal (NES) has been removed [24]. It has significantly increased mutation activity compared to wild-type AID without a NES [24]. Three successive transient transfections were performed before cells were screened for expression of mutant antibodies with increased avidity for HLA-A2.

Cells we started from stably expressed cell surface A2Ab and thus were able to bind tetramers comprising four HLA-A2 molecules. These cells were subjected to three successive transfections. We expected cells expressing higher avidity antibodies post-mutagenesis to be able to bind tetramers containing fewer HLA-A2 molecules. We thus sought to identify cells in the mutated polyclonal population using labeling with a tetramer made up of 3 HLA-A2 molecules and one B7 molecule (3A2/1B7). As shown in Fig. 3b, we were unable to detect any 3A2/1B7-labeled cells in the mutated polyclonal population by flow cytometry, while all cells expressing IgG were labeled with the initial tetramer (4A2) as expected.

We suspected that 3A2/1B7-labeled cells might be too rare to be detectable in the fraction of the mutated polyclonal population we tested, so we tried to enrich them before analysis. The mutated poylconal population was first incubated with the 3A2/1B7 tetramer coupled to APC, then subjected to positive selection using paramagnetic beads coupled to anti-APC antibodies. After magnetic enrichment, we observed a small proportion of cells clearly labeled by the 3A2/1B7 tetramer (Fig. 3c, left dot-plot). Notably, no such cells were detected when our protocol was carried out using A2Ab-expressing HEK 293 cells transfected with a hyperactive non-guided AID (Fig. 3c, middle dot-plot), or with guide RNAs alone (“mock”, Fig. 3c, right dot-plot). This first “positive” population (R1) was purified by cell sorting and expanded in vitro to yield population R1+ (> 95% pure). In marked contrast to the starting population, the R1+ population bound tetramers with just 3 HLA-A2 molecules (3A2/1B7, Fig. 3d, upper left dot-plot).

To complete a further round of mutagenesis/selection, we exposed the R1+ population to two successive transfections for mutagenesis using the same batch of sgRNAs as above, before selection was performed. This time we used a more stringent enrichment process with tetramers containing only one HLA-A2 molecule (1A2/3B7). A new population of tetramer positive cells was obtained (R2+), with a 2.2 fold increase in the 3A2/1B7 tetramer mean fluorescence intensity compared to R1+ (Fig. 3d, bottom left dot-plot). The R2+ population was also stained by tetramer 1A2/3B7, in marked contrast to R1+ cells (Fig. 3d, compare upper and lower right dot-plots). Each round of mutation and selection thus increases the avidity of the antibodies.

### Antibody sequence evolution during mutagenesis and selection rounds

**Fig. 4:**
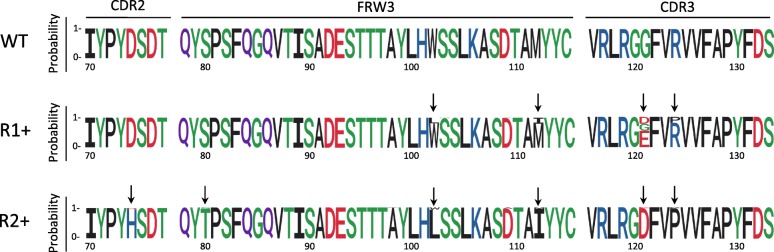
Web Logo representation of amino acid mutations in the A2Ab heavy chain. WT: starting sequence. R1+, R2+: sequences after one or two rounds of mutation/selection respectively. The height of each letter is proportional to the preference for that amino acid at that site, and letters are colored by amino-acid hydrophobicity. Residue positions are numbered starting from the first amino acid of the leader peptide of the heavy chain. Major mutation sites are indicated by arrows

As described above, we were unable to detect cells capable of binding to the 3A2/1B7 tetramer after one round of mutagenesis until we used magnetic enrichment. This enrichment generated the R1 population. FACS sorting of this population yielded the R1+ population capable of binding 3A2/1B7 tetramers and the R1- population incapable of binding this tetramer. We used next generation sequencing (NGS) to search for heavy chain sequences enriched in the R1+ population relative to the R1- population and which could contain mutations responsible for the increased affinity of the R1+ population antibodies. 30,000 randomly selected reads from each population were analyzed. Reads represented more than 50 times were placed into a read-specific cluster, while reads represented less than 50 times were grouped together in a category we termed “small clusters”. For the R1+ population, two large clusters representing together 42.5% of reads were detected, in addition to a third large cluster representing WT sequences (Table 1). Six other clusters representing together 5.2% of reads were also detected, together with numerous reads in the small cluster category. Seven of these eight non-WT clusters were clearly under-represented in the R1- population, where the WT cluster and small clusters predominated. Mutations observed in the seven clusters were located in the FRW3 and CDR3 regions (Fig. 4). They were often shared between different clusters, suggesting that they contribute to the increased affinity of R1+ population antibodies.

**Table 1:** CRISPR-X-mediated evolution of A2Ab: NGS analysis, round 1

R1			
Cluster name	mAb name	%R1+ (counts)	%R1- (counts)
G121E	C3.2	31.8 (9542)	0.3 (94)
WT	A2Ab	13.6 (4103)	52.6 (15788)
W102 L//M112I//G121D//R124P	C3.9	10.7 (3197)	0
G121E//V140 L		1.1 (340)	0
G121D	C3.3	1.1 (316)	0.3 (95)
S103 N//G121D	C3.5	0.9 (261)	0
W102 L//D109A//M112I//G121D//R124P		0.8 (239)	0
M112I//G121D//R124P		0.7 (209)	0
V140 L		0,6 (168)	1.5 (448)
R117S		0	0.5 (148)
Y114S		0	0.4 (124)
D109A		0	0.4 (119)
S103R		0	0.2 (67)
S108A		0	0.2 (66)
G137R		0	0.2 (61)
R119S		0	0.2 (60)
P60A		0	0.2 (54)
V123G		0	0.2 (54)
small clusters R1+ (number)	C3.4	38.7 (11625)	
small clusters R1- (number)			42.7 (12817)
total		100 (30000)	100 (30000)

That WT and small cluster sequences represent 52.6% of R1+ reads might seem surprising. However, in the HEK 293 cells subjected to mutagenesis, antibody genes are present on episomal vectors, with several vector copies per cell [25]. Cells selected with the 3A2/1B7 tetramer may contain only one gene copy with a mutation leading to an antibody of increased affinity. All the other copies could contain either no mutation or neutral or even deleterious mutations, yet they will be co-enriched with the copy carrying the affinity-increasing mutation.

The second round of mutation/selection led to a drastic decline in WT reads (from 13.6% for R1+, to 0% for R2+), while in the R2+ population a cluster representing nearly half of the NGS reads emerged, corresponding to HCs accumulating six mutated amino acids: D74H/S80 T/W102 L/M112I/G121D/R124P (Table 2, Fig. 4). Interestingly, the CDR2 D74H mutation was not detected in the R1+ population. Nine of the thirteen R2+ clusters (a cluster contains more than 50 reads of the cluster-specific sequence) differ only very slightly from this main sequence, underlining a strong convergence of most of the R2+ clusters. The W102, M112I, G121D and R124P mutations were already well represented in the R1+ population (Table 1). The second round of mutation/selection led to emergence of two new R2 + −specific mutations: D74H in the CDR2 and S80 T in the FRW3 region.

**Table 2:** CRISPR-X-mediated evolution of A2Ab: NGS analysis, round 2

R2			
Cluster name	mAb name	%R2+ (counts)	%R2- (counts)
D74H//S80 T//W102 L//M112I//G121D//R124P	C4.4	49.2 (14755)	9.2 (2756)
D74H//S80 T//W102 L//D109A//M112I//G121D//R124P		2.4 (733)	0.2 (73)
D74H//S80 T//M112I//G121D//R124P		2.2 (650)	0
G121E		1.7 (496)	38.9 (11670)
D74H//S80 T//F83S//W102 L//M112I//G121D//R124P		0.7 (223)	0.4 (112)
D74H//S80 T//A98P//W102 L//M112I//G121D//R124P		0.6 (182)	0
D74H//S80 T//W102 L//M112I//G121D//R124P//V140 L		0.6 (173)	0
D74H//W102 L//M112I//G121D//R124P	C4.18	0.5 (163)	0
G121D//R124P		0.2 (74)	0.9 (274)
D74H//S80 T//W102 L//S104 T//M112I//G121D//R124P		0.2 (72)	0
D74H//S80 T//W102 L//G121E		0.2 (68)	0
W102 L//M112I//G121D//R124P		0.2 (63)	4.2 (1247)
D74H//S80 T//W102 L//L105R//M112I//G121D//R124P		0.2 (59)	0
W52C//G121E		0	1.1 (333)
G121E//V140 L		0	0.7 (209)
R47S//R57H//G121E		0	0.7 (204)
W102 L//G121E		0	0.7 (203)
WT		0	0.6 (193)
M112I//G121D//R124P	A2Ab	0	0.5 (137)
W102 L//M112I//G121E		0	0.4 (128)
H101Q//G121E		0	0.4 (122)
I39M//H101Q//G121E		0	0.4 (119)
P60S//G121E		0	0,4 (81)
C41Y//G121E		0	0.2 (66)
I39M//G121E		0	0.2 (56)
W102C//G121E		0	0.2 (56)
small clusters R2+ (number)		41 (12289)	
small clusters R2- (number)			39.9 (11961)
total		100 (30000)	100 (30000)

### Characterization of evolved antibodies against HLA-A2

**Fig. 5:**
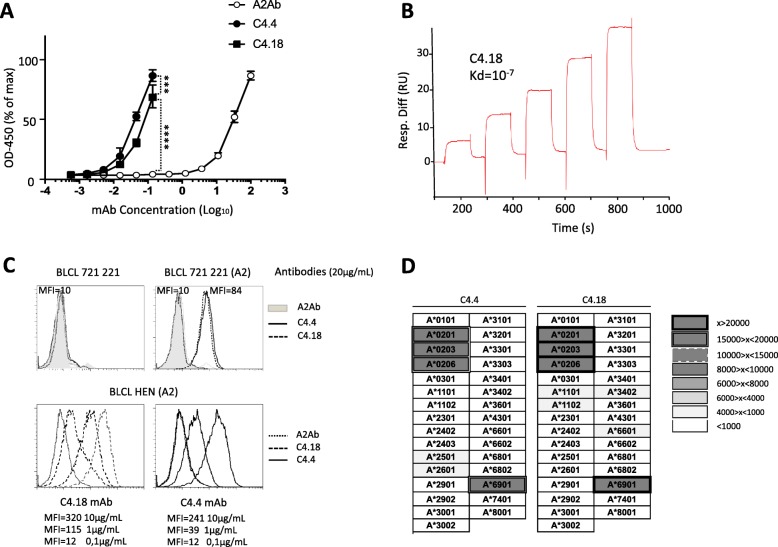
Characterization of evolved antibodies against HLA-A2. **a** ELISA dose-response curves of R2+ mutated mAbs C4.4 and C4.18 compared to A2Ab. Statistical significance was determined using a one-way ANOVA test followed by a Tukey’s multiple comparison post-test (*n* = 3, bars indicate standard deviations) (****: *p* < 0.0001; ***:*p* = 0,003). **b** The affinity of C4.18 was measured by surface plasmon resonance by flowing various concentrations of pp65-A2 complex over CM5 chip-bound C4.18. **c** Top panel: staining of 721.221 cells which either express HLA-A2 (721.221(A2)) or do not express it (721.221) by A2Ab, C4.4 and C4.18 at 20 μg/mL. MFI are indicated. Lower panel: dose response staining of A2Ab, C4.4 and C4.18 against BLCL HEN expressing HLA-A2. MFI obtained with various concentrations of C4.18 and C4.4 are indicated. **d** The specificity of mutated R2+ mAbs was assessed in a Luminex single antigen bead assay. Results are shown in terms of interval MFI. Positivity threshold was set at 1000

The R2+ antibodies C4.4 and C4.18 (Tables 1 and 2) were produced as recombinant proteins for comparison of their affinity and specificity to those of the initial A2Ab. As shown in Fig. 5a, C4.4 and C4.18 mAbs show clearly increased reactivity against HLA-A*02:01 compared to A2Ab in an ELISA. We next determined C4.18’s affinity for HLA-A*02:01 by SPR: Kd = 10^− 7^ (Fig. 5b). This is an almost two log increase over that of the initial A2Ab (Kd = 8 × 10^− 6^). We were unable to make enough C4.4 for SPR studies.

These results demonstrate that our matured antibodies bind with higher affinity to antigen than A2Ab in fully in vitro tests. But can they bind to antigen expressed on the surface of cells, a prerequisite for biological activity? The initial A2Ab was not of sufficient affinity to bind to two HLA-A2 expressing cell lines tested, 721.221 B cells made HLA-A2 positive by transfection (721.221(A2)), and naturally HLA-A2 expressing BLCL HEN. However, the increased affinity of C4.4 and C4.18 led to ready detection of such binding (Fig. 5c). Binding to 721.221(A2) B cells was HLA-A2 dependent, as no binding was observed to the parental HLA-A2 negative 721.221 B cells. A single HLA antigen flow bead assay analysis confirmed that C4.4 and C4.18 had higher affinity than A2Ab for HLA-A*02:01 and also showed a gain in specificity, as they had significantly less crossreactivity against other HLA-A alleles (compare Fig. 5d to Fig. 1c).

## Discussion

We show that starting from a low affinity antibody, CRISPR-X targeting of AID to antibody genes can be used to obtain affinity-matured human antibodies *in cellulo* in about 6 weeks. Thus we increased the affinity of a fully human anti-HLA-A*02:01 mAb to sufficient levels for biological activity and without loss of specificity in just 2 cycles of mutation/selection (each cycle consisting of several successive mutagenesis transfections prior to the selection steps). The low affinity antibody we started from was expressed by naive B cells. Our procedure thus mimics in vitro antibody maturation in secondary lymphoid organs, where naive B lymphocytes stimulated by Ag recognition via specific BCRs of limited affinity go on to generate receptors optimized for Ag recognition.

Using SHM for in vitro affinity maturation of antibodies is an attractive strategy and has been used previously in a variety of cell lines [2, 26–29]. Some recently described technologies to affinity-mature antibodies in vitro rely on the integration of a library of CDR3 domains using CRISPR Cas9 technology [30] or mutagenesis of only the most permissive CDR positions [31]. Prior to these approaches, the Bowers group pioneered the coupling of AID-induced somatic hypermutation with mammalian cell surface display in the easily transfectable HEK 293 cells for in vitro maturation of mAbs [15]. We have extended this latter approach to include specific targeting of AID to the immunoglobulin genes to be mutated using a combination of dCas9-AID fusions and specific guide RNAs. We have also introduced a magnetic enrichment step prior to FACS sorting of mutated cells to facilitate isolation of cells expressing higher affinity antibodies. These modifications proved necessary to obtain our affinity matured anti-HLA antibodies after only 2 rounds of mutation/selection. Indeed, we were unable to detect any cells carrying higher affinity antibodies when AID activity was not targeted to the Ig sequences, and we could only detect and isolate them after the first mutation round if magnetic enrichment preceded FACS sorting.

While this manuscript was in preparation, Liu et al. described a variety of diversifying base editors and showed that they retained their intrinsic nucleotide preferences when recruited to DNA as MS2 coat fusions [32]. They also demonstrated that it was possible to use diversifying base editors to affinity mature a previously studied murine anti-4-hydroxy-3-nitrophenylacetyl (NP) antibody called B1–8 [32]. The matured antibodies they obtained contained various mutations that had already been observed after subjecting B1–8 to SHM in a mouse in vivo immunization model. The effect of these point mutations was tested separately, and it was not clear whether any of their antibodies contained multiple mutations. In our study, we define previously unknown combination of mutations that are required to increase the affinity of a human antibody against HLA-A2, without loss of specificity. As might be expected, “beneficial” mutations could be found in the CDR2 and CDR3. Interestingly, CDR3 mutations appeared after the first round of mutation/selection, while CDR2 mutations only appeared after the second round. In addition to the CDR2 and CDR3 mutations, some mutations also appeared in the FRW3. In particular, the C4.18 mAb obtained after the second round of mutagenesis differs from the first round C3.9 mAb by only two additional mutated amino acids located in FRW3. This is interesting as antibody in vitro evolution studies have suggested that mutations leading to higher affinity often correspond to residues distant from the antigen binding site and that affinity maturation of antibodies occurs most effectively by changes in second sphere residues rather than contact residues [33, 34]. It is also interesting to note that increasing the affinity of our antibodies for HLA-A*02:01 also led to an increase in their specificity: they progressively lost their crossreactivity against non-HLA-A*02 alleles.

The progressive evolution of A2Ab we observed, with a gradual accumulation of combinations of mutations, is probably necessary for the maturation of the affinity of most antibodies. The combination of CDR and FRW mutations could result from CRISPR-X allowing simultaneous targeting of multiple sites all along the Ig variable sequence and potentially represents an important advantage over other recently described technologies limiting mutagenesis to the CDR3 [30] or to the most permissive CDR positions [31].

Our CRISPR-X based approach can readily be developed further to increase the potential for antibody diversification. We used the same 9 gRNAs for both rounds of mutagenesis. Further rounds of mutagenesis could be carried out using different gRNAs. The CRISPR-X approach using *S. pyogenes* dCas9 requires the presence of an NGG PAM immediately downstream from the gRNA binding site. Cas9 variants with relaxed PAM requirements could also be used in this approach, including the recently described variant using a PAM reduced to NG. This would lift almost all constraints on gRNA choice. We focused on mutating the Ig heavy chain gene alone, but both heavy and light chain genes were present in cells subjected to mutagenesis. We did not detect any light chain mutations after transfection of the heavy chain gRNAs (data not shown), demonstrating the specificity of the targeting approach. However, AID could be targeted simultaneously to both heavy and light chain genes by cotransfecting cells with a mixture of heavy and light chain gRNAs, increasing the diversification possibilities by association of mutated heavy and light chains in different combinations.

The A2Ab mAb used here served as an initial proof of concept for antibody maturation in vitro using CRISPR-X. However, the fully human mAbs specific for the HLA-A*02:01 allele we generated could have direct clinical applications, notably in the context of mismatch HLA-A2 organ transplantation. Two recent studies described the efficacy of anti-HLA-A2-specific CARs of murine origin in the control of graft rejection in animal models [35, 36]. Using fully human antibodies could be an important step forward for implementation of such strategies to humans. Furthermore, the availability of a series of mAbs of increasing affinity (derived from different rounds of mutation/selection) could be useful to study the impact of CAR affinity on biological activity and could also help to improve predictive algorythms for antibody maturation.

## Conclusions

We describe here a new approach for progressive and controlled antibody evolution. This procedure should allow us to obtain antibodies of high affinity and specificity against virtually any Ag, if available in a recombinant form, starting directly from circulating naïve B cells, which represent a vast pool of Ag-specific antibodies to tap into. Our approach may prove particularly useful when fully human antibodies are required: when first isolated from non-immunized individuals, they are often of insufficient affinity for therapeutic or research purposes. Many Ag of interest for the treatment of pathologies such as cancer are in this category and thus represent potential targets for this approach. In addition, our approach can be adapted to optimize antibody specificity by addition of a simple negative selection step to eliminate antibodies with undesired interactions. This could be useful for improving the specificity of currently existing murine, chimeric or humanized antibodies.

## Additional file

Additional file 1: **Table S1.** Isolation of human anti-HLA-A2 B lymphocytes from the PBMC of grafted patients.This table indicates the number of HLA-A2-specific B cells isolated from each donors.**Table S2.** Full nucleotide sequences.This table indicates the nucleotide sequences of the variable segments of the heavy and light chains of A2Ab and of the heavy chain of the various R1+ or R2+ mutants.**Table S3.** gRNA sequences binding to the Ig gene sense (s) or antisense (as) strands.This table indicates the nucleotide sequence of the gRNAs which are numbered according to their position from the ATG (A corresponding to nucleotide number 1) of the A2Ab variable heavy chain sequence (see Additional file 1: **Table S2**). (DOCX 51 kb)

## Abbreviations

Ag: Antigen
APC: Allophycocyanin
BCR: B-cell receptor
BV: Brillant violet
CDR: Complementarity determining region
FRW: Framework region
HC: Heavy chain
LC: Light chain
mAbs: Monoclonal antibodies
PBMC: Peripheral blood mononuclear cells
PE: Phycoerythrin
pMHC: Peptide-major histocompatibility complex
SPR: Surface plasmon resonance

## References

[1] Schatz DG, Ji Y (2011). Recombination centres and the orchestration of V(D)J recombination. Nat Rev Immunol.

[2] Martin A, Scharff MD (2002). Somatic hypermutation of the AID transgene in B and non-B cells. Proc Natl Acad Sci U S A.

[3] Muramatsu M, Kinoshita K, Fagarasan S, Yamada S, Shinkai Y, Honjo T (2000). Class switch recombination and hypermutation require activation-induced cytidine deaminase (AID), a potential RNA editing enzyme. Cell.

[4] Williams SC, Frippiat JP, Tomlinson IM, Ignatovich O, Lefranc MP, Winter G (1996). Sequence and evolution of the human germline V lambda repertoire. J Mol Biol.

[5] Calis JJ, Rosenberg BR (2014). Characterizing immune repertoires by high throughput sequencing: strategies and applications. Trends Immunol.

[6] Corti D, Langedijk JP, Hinz A, Seaman MS, Vanzetta F, Fernandez-Rodriguez BM, Silacci C, Pinna D, Jarrossay D, Balla-Jhagjhoorsingh S (2010). Analysis of memory B cell responses and isolation of novel monoclonal antibodies with neutralizing breadth from HIV-1-infected individuals. PLoS One.

[7] Corti D, Voss J, Gamblin SJ, Codoni G, Macagno A, Jarrossay D, Vachieri SG, Pinna D, Minola A, Vanzetta F (2011). A neutralizing antibody selected from plasma cells that binds to group 1 and group 2 influenza a hemagglutinins. Science.

[8] Devilder MC, Moyon M, Saulquin X, Gautreau-Rolland L. Generation of discriminative human monoclonal antibodies from rare antigen-specific B cells circulating in blood. J Vis Exp. 2018;(132). 10.3791/56508 PMC5912359 29443062

[9] Franz B, May KF, Dranoff G, Wucherpfennig K (2011). Ex vivo characterization and isolation of rare memory B cells with antigen tetramers. Blood.

[10] Ouisse LH, Gautreau-Rolland L, Devilder MC, Osborn M, Moyon M, Visentin J, Halary F, Bruggemann M, Buelow R, Anegon I (2017). Antigen-specific single B cell sorting and expression-cloning from immunoglobulin humanized rats: a rapid and versatile method for the generation of high affinity and discriminative human monoclonal antibodies. BMC Biotechnol.

[11] Hoogenboom HR (2005). Selecting and screening recombinant antibody libraries. Nat Biotechnol.

[12] Akamatsu Y, Pakabunto K, Xu Z, Zhang Y, Tsurushita N (2007). Whole IgG surface display on mammalian cells: application to isolation of neutralizing chicken monoclonal anti-IL-12 antibodies. J Immunol Methods.

[13] Al-Qaisi TS, Su YC, Roffler SR (2018). Transient AID expression for in situ mutagenesis with improved cellular fitness. Sci Rep.

[14] An L, Chen C, Luo R, Zhao Y, Hang H (2018). Activation-induced cytidine deaminase aided in vitro antibody evolution. Methods Mol Biol.

[15] Bowers PM, Horlick RA, Neben TY, Toobian RM, Tomlinson GL, Dalton JL, Jones HA, Chen A, Altobell L, Zhang X (2011). Coupling mammalian cell surface display with somatic hypermutation for the discovery and maturation of human antibodies. Proc Natl Acad Sci U S A.

[16] Ho M, Nagata S, Pastan I (2006). Isolation of anti-CD22 Fv with high affinity by Fv display on human cells. Proc Natl Acad Sci U S A.

[17] Ho M, Pastan I (2009). Display and selection of scFv antibodies on HEK-293T cells. Methods Mol Biol.

[18] McConnell AD, Do M, Neben TY, Spasojevic V, MacLaren J, Chen AP, Altobell L, Macomber JL, Berkebile AD, Horlick RA (2012). High affinity humanized antibodies without making hybridomas; immunization paired with mammalian cell display and in vitro somatic hypermutation. PLoS One.

[19] Su YC, Al-Qaisi TS, Tung HY, Cheng TL, Chuang KH, Chen BM, Roffler SR (2014). Mimicking the germinal center reaction in hybridoma cells to isolate temperature-selective anti-PEG antibodies. mAbs.

[20] Wang L, Jackson WC, Steinbach PA, Tsien RY (2004). Evolution of new nonantibody proteins via iterative somatic hypermutation. Proc Natl Acad Sci U S A.

[21] Hwang JK, Alt FW, Yeap LS: Related Mechanisms of Antibody Somatic Hypermutation and Class Switch Recombination. Microbiol Spectr 2015, 3(1):MDNA3–0037-2014. 10.1128/microbiolspec.MDNA3-0037-2014 PMC4481323 26104555

[22] Hess GT, Tycko J, Yao D, Bassik MC (2017). Methods and applications of CRISPR-Mediated Base editing in eukaryotic genomes. Mol Cell.

[23] Rees HA, Liu DR (2018). Base editing: precision chemistry on the genome and transcriptome of living cells. Nat Rev Genet.

[24] Hess GT, Fresard L, Han K, Lee CH, Li A, Cimprich KA, Montgomery SB, Bassik MC (2016). Directed evolution using dCas9-targeted somatic hypermutation in mammalian cells. Nat Methods.

[25] Yates JL, Warren N, Sugden B (1985). Stable replication of plasmids derived from Epstein-Barr virus in various mammalian cells. Nature.

[26] Cumbers SJ, Williams GT, Davies SL, Grenfell RL, Takeda S, Batista FD, Sale JE, Neuberger MS (2002). Generation and iterative affinity maturation of antibodies in vitro using hypermutating B-cell lines. Nat Biotechnol.

[27] Delker RK, Fugmann SD, Papavasiliou FN (2009). A coming-of-age story: activation-induced cytidine deaminase turns 10. Nat Immunol.

[28] Maul RW, Gearhart PJ (2010). AID and somatic hypermutation. Adv Immunol.

[29] Seo H, Hashimoto S, Tsuchiya K, Lin W, Shibata T, Ohta K (2006). An ex vivo method for rapid generation of monoclonal antibodies (ADLib system). Nat Protoc.

[30] Mason DM, Weber CR, Parola C, Meng SM, Greiff V, Kelton WJ, Reddy ST (2018). High-throughput antibody engineering in mammalian cells by CRISPR/Cas9-mediated homology-directed mutagenesis. Nucleic Acids Res.

[31] Tiller KE, Chowdhury R, Li T, Ludwig SD, Sen S, Maranas CD, Tessier PM (2017). Facile affinity maturation of antibody variable domains using natural diversity mutagenesis. Front Immunol.

[32] Liu LD, Huang M, Dai P, Liu T, Fan S, Cheng X, Zhao Y, Yeap LS, Meng FL: Intrinsic nucleotide preference of Diversifying Base editors guides antibody ex vivo affinity maturation. Cell Rep 2018, 25(4):884–892 e883. 10.1016/j.celrep.2018.09.090 30355495

[33] Boder ET, Midelfort KS, Wittrup KD (2000). Directed evolution of antibody fragments with monovalent femtomolar antigen-binding affinity. Proc Natl Acad Sci U S A.

[34] Persson H, Kirik U, Thornqvist L, Greiff L, Levander F, Ohlin M (2018). In vitro evolution of antibodies inspired by in vivo evolution. Front Immunol.

[35] MacDonald KG, Hoeppli RE, Huang Q, Gillies J, Luciani DS, Orban PC, Broady R, Levings MK (2016). Alloantigen-specific regulatory T cells generated with a chimeric antigen receptor. J Clin Invest.

[36] Noyan F, Zimmermann K, Hardtke-Wolenski M, Knoefel A, Schulde E, Geffers R, Hust M, Huehn J, Galla M, Morgan M (2017). Prevention of allograft rejection by use of regulatory T cells with an MHC-specific chimeric antigen receptor. Am J Transplant.

