# Supplementary material for: Ex vivo evolution of human antibodies by CRISPR-X: from a naive B cell repertoire to affinity matured antibodies
Source: BMC Biotechnol. 2019 Feb 18;19:14. doi: 10.1186/s12896-019-0504-z (PMC6378725; doi:10.1186/s12896-019-0504-z)
Supplement: Supplementary file 1 — Table S1. Isolation of human anti-HLA-A2 B lymphocytes from the PBMC of grafted patients.This table indicates the number of HLA-A2-specific B cells isolated from each donors.Table S2. Full nucleotide sequences.This table indicates the nucleotide sequences of the variable segments of the heavy and light chains of A2Ab and of the heavy chain of the various R1+ or R2+ mutants.Table S3. gRNA sequences binding to the Ig gene sense (s) or antisense (as) strands.This table indicates the nucleotide sequence of the gRNAs which are numbered according to their position from the ATG (A corresponding to nucleotide number 1) of the A2Ab variable heavy chain sequence (see Additional file 1: Table S2). (DOCX 51 kb) [file 12896_2019_504_MOESM1_ESM.docx]

Table S1: Isolation of human anti-HLA-A2 B lymphocytes from the PBMC of grafted patients.

| Donor | Anti HLA-A2 serology | Number of PBMC analyzed (million) | Number of HLA-A2 specific B cells isolated |
| --- | --- | --- | --- |
| H1 | Negative | 10 | 1 |
| H2 | Negative | 10 | 2 |
| NO | Negative | 10 | 4 |

Table S2 : Full nucleotide sequences (wt and mutants)

Wt-A2Ab

|  |  |  |  |
| --- | --- | --- | --- |
| chain |  |  | 5'-3' DNA sequence |
| heavy |  |  | ATGGGATGGTCATGTATCATCCTTTTTCTAGTAGCAACTGCAACCGGTGTACATTCTGAGGTGCAGCTGGTGGAGTCTGGAGCAGAGGTGAAAAAGCCGGGGGAGTCTCTGAAGATCTCCTGTAAGGCCTCTGGATACAGGTTCACCAACTACTGGATCGGCTGGGTGCGCCAGATGCCCGGGAAAGGCCTGGAGTGGATGGGGATCATCTATCCTTATGACTCTGATACCCAATACAGCCCGTCCTTCCAAGGCCAGGTCACCATCTCAGCCGACGAGTCCACCACCACCGCCTACCTGCACTGGAGCAGCCTGAAGGCCTCGGACACCGCCATGTATTACTGTGTGAGACTCAGGGGAGGTTTTGTCCGAGTGGTGTTCGCCCCCTACTTTGACTCCTGGGGCCAGGGAACTCTGGTCACC |
| Light |  |  | ATGGGATGGTCATGTATCATCCTTTTTCTAGTAGCAACTGCAACCGGTTCCTGGGCCCAGTCTGCCCTGACTCAGCCTCGCTCAGTGTCCGGGTCTCCTGGACAGTCAGTCACCATCTCCTGCACTGGAAGCAGAAGTGATGCTCATACTTTTAACTATGTCTCCTGGTACCAACAACACCCAGGCAAAGCCCCCAAACTCATGATTTGTGATGTCAATCAGCGGCCCTCAGGGGTCCCTGATCGCTTCTCTGGCTCCAAGTCTGGCGACGCGGCCTCCCTGACCATCTCTGGGCTCCAGGCTGAAGATGAGGCTGATTATTACTGCTTCTCATATGATGCCAACTACACTTTAGGGGTCTTCGGAACTGGGACCAAG |

| R1 |  |  |  |
| --- | --- | --- | --- |
|  |  |  |  |
| Cluster name |  | Nucleotide mutations | 5'-3' DNA sequence |
|  |  |  |  |
| G121E |  | G362A//T363A | ATGGGATGGTCATGTATCATCCTTTTTCTAGTAGCAACTGCAACCGGTGTACATTCTGAGGTGCAGCTGGTGGAGTCTGGAGCAGAGGTGAAAAAGCCGGGGGAGTCTCTGAAGATCTCCTGTAAGGCCTCTGGATACAGGTTCACCAACTACTGGATCGGCTGGGTGCGCCAGATGCCCGGGAAAGGCCTGGAGTGGATGGGGATCATCTATCCTTATGACTCTGATACCCAATACAGCCCGTCCTTCCAAGGCCAGGTCACCATCTCAGCCGACGAGTCCACCACCACCGCCTACCTGCACTGGAGCAGCCTGAAGGCCTCGGACACCGCCATGTATTACTGTGTGAGACTCAGGGGAGAATTTGTCCGAGTGGTGTTCGCCCCCTACTTTGACTCCTGGGGCCAGGGAACTCTGGTCACC |
| W102L//M112I//G121D//R124P | | G305T//G336A//G362A//G371C | ATGGGATGGTCATGTATCATCCTTTTTCTAGTAGCAACTGCAACCGGTGTACATTCTGAGGTGCAGCTGGTGGAGTCTGGAGCAGAGGTGAAAAAGCCGGGGGAGTCTCTGAAGATCTCCTGTAAGGCCTCTGGATACAGGTTCACCAACTACTGGATCGGCTGGGTGCGCCAGATGCCCGGGAAAGGCCTGGAGTGGATGGGGATCATCTATCCTTATGACTCTGATACCCAATACAGCCCGTCCTTCCAAGGCCAGGTCACCATCTCAGCCGACGAGTCCACCACCACCGCCTACCTGCACTTGAGCAGCCTGAAGGCCTCGGACACCGCCATATATTACTGTGTGAGACTCAGGGGAGATTTTGTCCCAGTGGTGTTCGCCCCCTACTTTGACTCCTGGGGCCAGGGAACTCTGGTCACC |
| G121E//V140L |  | G362A//T363A//G418C | ATGGGATGGTCATGTATCATCCTTTTTCTAGTAGCAACTGCAACCGGTGTACATTCTGAGGTGCAGCTGGTGGAGTCTGGAGCAGAGGTGAAAAAGCCGGGGGAGTCTCTGAAGATCTCCTGTAAGGCCTCTGGATACAGGTTCACCAACTACTGGATCGGCTGGGTGCGCCAGATGCCCGGGAAAGGCCTGGAGTGGATGGGGATCATCTATCCTTATGACTCTGATACCCAATACAGCCCGTCCTTCCAAGGCCAGGTCACCATCTCAGCCGACGAGTCCACCACCACCGCCTACCTGCACTGGAGCAGCCTGAAGGCCTCGGACACCGCCATGTATTACTGTGTGAGACTCAGGGGAGAATTTGTCCGAGTGGTGTTCGCCCCCTACTTTGACTCCTGGGGCCAGGGAACTCTGCTCACC |
| G121D |  | G362A | ATGGGATGGTCATGTATCATCCTTTTTCTAGTAGCAACTGCAACCGGTGTACATTCTGAGGTGCAGCTGGTGGAGTCTGGAGCAGAGGTGAAAAAGCCGGGGGAGTCTCTGAAGATCTCCTGTAAGGCCTCTGGATACAGGTTCACCAACTACTGGATCGGCTGGGTGCGCCAGATGCCCGGGAAAGGCCTGGAGTGGATGGGGATCATCTATCCTTATGACTCTGATACCCAATACAGCCCGTCCTTCCAAGGCCAGGTCACCATCTCAGCCGACGAGTCCACCACCACCGCCTACCTGCACTGGAGCAGCCTGAAGGCCTCGGACACCGCCATGTATTACTGTGTGAGACTCAGGGGAGATTTTGTCCGAGTGGTGTTCGCCCCCTACTTTGACTCCTGGGGCCAGGGAACTCTGGTCACC |
| S103N//G121D |  | G308A//G357A//G362A | ATGGGATGGTCATGTATCATCCTTTTTCTAGTAGCAACTGCAACCGGTGTACATTCTGAGGTGCAGCTGGTGGAGTCTGGAGCAGAGGTGAAAAAGCCGGGGGAGTCTCTGAAGATCTCCTGTAAGGCCTCTGGATACAGGTTCACCAACTACTGGATCGGCTGGGTGCGCCAGATGCCCGGGAAAGGCCTGGAGTGGATGGGGATCATCTATCCTTATGACTCTGATACCCAATACAGCCCGTCCTTCCAAGGCCAGGTCACCATCTCAGCCGACGAGTCCACCACCACCGCCTACCTGCACTGGAACAGCCTGAAGGCCTCGGACACCGCCATGTATTACTGTGTGAGACTCAGAGGAGATTTTGTCCGAGTGGTGTTCGCCCCCTACTTTGACTCCTGGGGCCAGGGAACTCTGGTCACC |
| W102L//D109A//M112I//G121D//R124P | | G305T//A326C//G336A//G362A//G371C | ATGGGATGGTCATGTATCATCCTTTTTCTAGTAGCAACTGCAACCGGTGTACATTCTGAGGTGCAGCTGGTGGAGTCTGGAGCAGAGGTGAAAAAGCCGGGGGAGTCTCTGAAGATCTCCTGTAAGGCCTCTGGATACAGGTTCACCAACTACTGGATCGGCTGGGTGCGCCAGATGCCCGGGAAAGGCCTGGAGTGGATGGGGATCATCTATCCTTATGACTCTGATACCCAATACAGCCCGTCCTTCCAAGGCCAGGTCACCATCTCAGCCGACGAGTCCACCACCACCGCCTACCTGCACTTGAGCAGCCTGAAGGCCTCGGCCACCGCCATATATTACTGTGTGAGACTCAGGGGAGATTTTGTCCCAGTGGTGTTCGCCCCCTACTTTGACTCCTGGGGCCAGGGAACTCTGGTCACC |
| M112I//G121D//R124P |  | G336A//G362A//G371C | ATGGGATGGTCATGTATCATCCTTTTTCTAGTAGCAACTGCAACCGGTGTACATTCTGAGGTGCAGCTGGTGGAGTCTGGAGCAGAGGTGAAAAAGCCGGGGGAGTCTCTGAAGATCTCCTGTAAGGCCTCTGGATACAGGTTCACCAACTACTGGATCGGCTGGGTGCGCCAGATGCCCGGGAAAGGCCTGGAGTGGATGGGGATCATCTATCCTTATGACTCTGATACCCAATACAGCCCGTCCTTCCAAGGCCAGGTCACCATCTCAGCCGACGAGTCCACCACCACCGCCTACCTGCACTGGAGCAGCCTGAAGGCCTCGGACACCGCCATATATTACTGTGTGAGACTCAGGGGAGATTTTGTCCCAGTGGTGTTCGCCCCCTACTTTGACTCCTGGGGCCAGGGAACTCTGGTCACC |
| V140L |  | G418C | ATGGGATGGTCATGTATCATCCTTTTTCTAGTAGCAACTGCAACCGGTGTACATTCTGAGGTGCAGCTGGTGGAGTCTGGAGCAGAGGTGAAAAAGCCGGGGGAGTCTCTGAAGATCTCCTGTAAGGCCTCTGGATACAGGTTCACCAACTACTGGATCGGCTGGGTGCGCCAGATGCCCGGGAAAGGCCTGGAGTGGATGGGGATCATCTATCCTTATGACTCTGATACCCAATACAGCCCGTCCTTCCAAGGCCAGGTCACCATCTCAGCCGACGAGTCCACCACCACCGCCTACCTGCACTGGAGCAGCCTGAAGGCCTCGGACACCGCCATGTATTACTGTGTGAGACTCAGGGGAGGTTTTGTCCGAGTGGTGTTCGCCCCCTACTTTGACTCCTGGGGCCAGGGAACTCTGCTCACC |
| R117S |  | A351C | ATGGGATGGTCATGTATCATCCTTTTTCTAGTAGCAACTGCAACCGGTGTACATTCTGAGGTGCAGCTGGTGGAGTCTGGAGCAGAGGTGAAAAAGCCGGGGGAGTCTCTGAAGATCTCCTGTAAGGCCTCTGGATACAGGTTCACCAACTACTGGATCGGCTGGGTGCGCCAGATGCCCGGGAAAGGCCTGGAGTGGATGGGGATCATCTATCCTTATGACTCTGATACCCAATACAGCCCGTCCTTCCAAGGCCAGGTCACCATCTCAGCCGACGAGTCCACCACCACCGCCTACCTGCACTGGAGCAGCCTGAAGGCCTCGGACACCGCCATGTATTACTGTGTGAGCCTCAGGGGAGGTTTTGTCCGAGTGGTGTTCGCCCCCTACTTTGACTCCTGGGGCCAGGGAACTCTGGTCACC |
| Y114S |  | A341C | ATGGGATGGTCATGTATCATCCTTTTTCTAGTAGCAACTGCAACCGGTGTACATTCTGAGGTGCAGCTGGTGGAGTCTGGAGCAGAGGTGAAAAAGCCGGGGGAGTCTCTGAAGATCTCCTGTAAGGCCTCTGGATACAGGTTCACCAACTACTGGATCGGCTGGGTGCGCCAGATGCCCGGGAAAGGCCTGGAGTGGATGGGGATCATCTATCCTTATGACTCTGATACCCAATACAGCCCGTCCTTCCAAGGCCAGGTCACCATCTCAGCCGACGAGTCCACCACCACCGCCTACCTGCACTGGAGCAGCCTGAAGGCCTCGGACACCGCCATGTATTCCTGTGTGAGACTCAGGGGAGGTTTTGTCCGAGTGGTGTTCGCCCCCTACTTTGACTCCTGGGGCCAGGGAACTCTGGTCACC |
| D109A |  | A326C | ATGGGATGGTCATGTATCATCCTTTTTCTAGTAGCAACTGCAACCGGTGTACATTCTGAGGTGCAGCTGGTGGAGTCTGGAGCAGAGGTGAAAAAGCCGGGGGAGTCTCTGAAGATCTCCTGTAAGGCCTCTGGATACAGGTTCACCAACTACTGGATCGGCTGGGTGCGCCAGATGCCCGGGAAAGGCCTGGAGTGGATGGGGATCATCTATCCTTATGACTCTGATACCCAATACAGCCCGTCCTTCCAAGGCCAGGTCACCATCTCAGCCGACGAGTCCACCACCACCGCCTACCTGCACTGGAGCAGCCTGAAGGCCTCGGCCACCGCCATGTATTACTGTGTGAGACTCAGGGGAGGTTTTGTCCGAGTGGTGTTCGCCCCCTACTTTGACTCCTGGGGCCAGGGAACTCTGGTCACC |
| S103R |  | A307C | ATGGGATGGTCATGTATCATCCTTTTTCTAGTAGCAACTGCAACCGGTGTACATTCTGAGGTGCAGCTGGTGGAGTCTGGAGCAGAGGTGAAAAAGCCGGGGGAGTCTCTGAAGATCTCCTGTAAGGCCTCTGGATACAGGTTCACCAACTACTGGATCGGCTGGGTGCGCCAGATGCCCGGGAAAGGCCTGGAGTGGATGGGGATCATCTATCCTTATGACTCTGATACCCAATACAGCCCGTCCTTCCAAGGCCAGGTCACCATCTCAGCCGACGAGTCCACCACCACCGCCTACCTGCACTGGCGCAGCCTGAAGGCCTCGGACACCGCCATGTATTACTGTGTGAGACTCAGGGGAGGTTTTGTCCGAGTGGTGTTCGCCCCCTACTTTGACTCCTGGGGCCAGGGAACTCTGGTCACC |
| S108A |  | T322G | ATGGGATGGTCATGTATCATCCTTTTTCTAGTAGCAACTGCAACCGGTGTACATTCTGAGGTGCAGCTGGTGGAGTCTGGAGCAGAGGTGAAAAAGCCGGGGGAGTCTCTGAAGATCTCCTGTAAGGCCTCTGGATACAGGTTCACCAACTACTGGATCGGCTGGGTGCGCCAGATGCCCGGGAAAGGCCTGGAGTGGATGGGGATCATCTATCCTTATGACTCTGATACCCAATACAGCCCGTCCTTCCAAGGCCAGGTCACCATCTCAGCCGACGAGTCCACCACCACCGCCTACCTGCACTGGAGCAGCCTGAAGGCCGCGGACACCGCCATGTATTACTGTGTGAGACTCAGGGGAGGTTTTGTCCGAGTGGTGTTCGCCCCCTACTTTGACTCCTGGGGCCAGGGAACTCTGGTCACC |
| G137R |  | G409C | ATGGGATGGTCATGTATCATCCTTTTTCTAGTAGCAACTGCAACCGGTGTACATTCTGAGGTGCAGCTGGTGGAGTCTGGAGCAGAGGTGAAAAAGCCGGGGGAGTCTCTGAAGATCTCCTGTAAGGCCTCTGGATACAGGTTCACCAACTACTGGATCGGCTGGGTGCGCCAGATGCCCGGGAAAGGCCTGGAGTGGATGGGGATCATCTATCCTTATGACTCTGATACCCAATACAGCCCGTCCTTCCAAGGCCAGGTCACCATCTCAGCCGACGAGTCCACCACCACCGCCTACCTGCACTGGAGCAGCCTGAAGGCCTCGGACACCGCCATGTATTACTGTGTGAGACTCAGGGGAGGTTTTGTCCGAGTGGTGTTCGCCCCCTACTTTGACTCCTGGGGCCAGCGAACTCTGGTCACC |
| R119S |  | G357C | ATGGGATGGTCATGTATCATCCTTTTTCTAGTAGCAACTGCAACCGGTGTACATTCTGAGGTGCAGCTGGTGGAGTCTGGAGCAGAGGTGAAAAAGCCGGGGGAGTCTCTGAAGATCTCCTGTAAGGCCTCTGGATACAGGTTCACCAACTACTGGATCGGCTGGGTGCGCCAGATGCCCGGGAAAGGCCTGGAGTGGATGGGGATCATCTATCCTTATGACTCTGATACCCAATACAGCCCGTCCTTCCAAGGCCAGGTCACCATCTCAGCCGACGAGTCCACCACCACCGCCTACCTGCACTGGAGCAGCCTGAAGGCCTCGGACACCGCCATGTATTACTGTGTGAGACTCAGCGGAGGTTTTGTCCGAGTGGTGTTCGCCCCCTACTTTGACTCCTGGGGCCAGGGAACTCTGGTCACC |
| P60A |  | C178G | ATGGGATGGTCATGTATCATCCTTTTTCTAGTAGCAACTGCAACCGGTGTACATTCTGAGGTGCAGCTGGTGGAGTCTGGAGCAGAGGTGAAAAAGCCGGGGGAGTCTCTGAAGATCTCCTGTAAGGCCTCTGGATACAGGTTCACCAACTACTGGATCGGCTGGGTGCGCCAGATGGCCGGGAAAGGCCTGGAGTGGATGGGGATCATCTATCCTTATGACTCTGATACCCAATACAGCCCGTCCTTCCAAGGCCAGGTCACCATCTCAGCCGACGAGTCCACCACCACCGCCTACCTGCACTGGAGCAGCCTGAAGGCCTCGGACACCGCCATGTATTACTGTGTGAGACTCAGGGGAGGTTTTGTCCGAGTGGTGTTCGCCCCCTACTTTGACTCCTGGGGCCAGGGAACTCTGGTCACC |
| V123G |  | T368G | ATGGGATGGTCATGTATCATCCTTTTTCTAGTAGCAACTGCAACCGGTGTACATTCTGAGGTGCAGCTGGTGGAGTCTGGAGCAGAGGTGAAAAAGCCGGGGGAGTCTCTGAAGATCTCCTGTAAGGCCTCTGGATACAGGTTCACCAACTACTGGATCGGCTGGGTGCGCCAGATGCCCGGGAAAGGCCTGGAGTGGATGGGGATCATCTATCCTTATGACTCTGATACCCAATACAGCCCGTCCTTCCAAGGCCAGGTCACCATCTCAGCCGACGAGTCCACCACCACCGCCTACCTGCACTGGAGCAGCCTGAAGGCCTCGGACACCGCCATGTATTACTGTGTGAGACTCAGGGGAGGTTTTGGCCGAGTGGTGTTCGCCCCCTACTTTGACTCCTGGGGCCAGGGAACTCTGGTCACC |

|  |  |  |  |
| --- | --- | --- | --- |
|  |  |  |  |

| R2 |  |  |  |
| --- | --- | --- | --- |
|  |  |  |  |
| Cluster name |  |  |  |
|  |  |  |  |
| D74H//S80T//W102L//M112I//G121D//R124P | | G204A//G220C//G239C//G305T//G336A//G362A//G371C | ATGGGATGGTCATGTATCATCCTTTTTCTAGTAGCAACTGCAACCGGTGTACATTCTGAGGTGCAGCTGGTGGAGTCTGGAGCAGAGGTGAAAAAGCCGGGGGAGTCTCTGAAGATCTCCTGTAAGGCCTCTGGATACAGGTTCACCAACTACTGGATCGGCTGGGTGCGCCAGATGCCCGGGAAAGGCCTGGAGTGGATGGGAATCATCTATCCTTATCACTCTGATACCCAATACACCCCGTCCTTCCAAGGCCAGGTCACCATCTCAGCCGACGAGTCCACCACCACCGCCTACCTGCACTTGAGCAGCCTGAAGGCCTCGGACACCGCCATATATTACTGTGTGAGACTCAGGGGAGATTTTGTCCCAGTGGTGTTCGCCCCCTACTTTGACTCCTGGGGCCAGGGAACTCTGGTCACC |
| D74H//S80T//W102L//D109A//M112I//G121D//R124P | | G204A//G220C//G239C//G305T//A326C//G336A//G362A//G371C | ATGGGATGGTCATGTATCATCCTTTTTCTAGTAGCAACTGCAACCGGTGTACATTCTGAGGTGCAGCTGGTGGAGTCTGGAGCAGAGGTGAAAAAGCCGGGGGAGTCTCTGAAGATCTCCTGTAAGGCCTCTGGATACAGGTTCACCAACTACTGGATCGGCTGGGTGCGCCAGATGCCCGGGAAAGGCCTGGAGTGGATGGGAATCATCTATCCTTATCACTCTGATACCCAATACACCCCGTCCTTCCAAGGCCAGGTCACCATCTCAGCCGACGAGTCCACCACCACCGCCTACCTGCACTTGAGCAGCCTGAAGGCCTCGGCCACCGCCATATATTACTGTGTGAGACTCAGGGGAGATTTTGTCCCAGTGGTGTTCGCCCCCTACTTTGACTCCTGGGGCCAGGGAACTCTGGTCACC |
| D74H//S80T//M112I//G121D//R124P | | G204A//G220C//G239C//G336A//G362A//G371C | ATGGGATGGTCATGTATCATCCTTTTTCTAGTAGCAACTGCAACCGGTGTACATTCTGAGGTGCAGCTGGTGGAGTCTGGAGCAGAGGTGAAAAAGCCGGGGGAGTCTCTGAAGATCTCCTGTAAGGCCTCTGGATACAGGTTCACCAACTACTGGATCGGCTGGGTGCGCCAGATGCCCGGGAAAGGCCTGGAGTGGATGGGAATCATCTATCCTTATCACTCTGATACCCAATACACCCCGTCCTTCCAAGGCCAGGTCACCATCTCAGCCGACGAGTCCACCACCACCGCCTACCTGCACTGGAGCAGCCTGAAGGCCTCGGACACCGCCATATATTACTGTGTGAGACTCAGGGGAGATTTTGTCCCAGTGGTGTTCGCCCCCTACTTTGACTCCTGGGGCCAGGGAACTCTGGTCACC |
| G121E |  | G362A//T363A | ATGGGATGGTCATGTATCATCCTTTTTCTAGTAGCAACTGCAACCGGTGTACATTCTGAGGTGCAGCTGGTGGAGTCTGGAGCAGAGGTGAAAAAGCCGGGGGAGTCTCTGAAGATCTCCTGTAAGGCCTCTGGATACAGGTTCACCAACTACTGGATCGGCTGGGTGCGCCAGATGCCCGGGAAAGGCCTGGAGTGGATGGGGATCATCTATCCTTATGACTCTGATACCCAATACAGCCCGTCCTTCCAAGGCCAGGTCACCATCTCAGCCGACGAGTCCACCACCACCGCCTACCTGCACTGGAGCAGCCTGAAGGCCTCGGACACCGCCATGTATTACTGTGTGAGACTCAGGGGAGAATTTGTCCGAGTGGTGTTCGCCCCCTACTTTGACTCCTGGGGCCAGGGAACTCTGGTCACC |
| D74H//S80T//F83S//W102L//M112I//G121D//R124P | | G204A//G220C//G239C//T248C//G305T//G336A//G362A//G371C | ATGGGATGGTCATGTATCATCCTTTTTCTAGTAGCAACTGCAACCGGTGTACATTCTGAGGTGCAGCTGGTGGAGTCTGGAGCAGAGGTGAAAAAGCCGGGGGAGTCTCTGAAGATCTCCTGTAAGGCCTCTGGATACAGGTTCACCAACTACTGGATCGGCTGGGTGCGCCAGATGCCCGGGAAAGGCCTGGAGTGGATGGGAATCATCTATCCTTATCACTCTGATACCCAATACACCCCGTCCTCCCAAGGCCAGGTCACCATCTCAGCCGACGAGTCCACCACCACCGCCTACCTGCACTTGAGCAGCCTGAAGGCCTCGGACACCGCCATATATTACTGTGTGAGACTCAGGGGAGATTTTGTCCCAGTGGTGTTCGCCCCCTACTTTGACTCCTGGGGCCAGGGAACTCTGGTCACC |
| D74H//S80T//A98P//W102L//M112I//G121D//R124P | | G204A//G220C//G239C//G292C//G305T//G336A//G362A//G371C | ATGGGATGGTCATGTATCATCCTTTTTCTAGTAGCAACTGCAACCGGTGTACATTCTGAGGTGCAGCTGGTGGAGTCTGGAGCAGAGGTGAAAAAGCCGGGGGAGTCTCTGAAGATCTCCTGTAAGGCCTCTGGATACAGGTTCACCAACTACTGGATCGGCTGGGTGCGCCAGATGCCCGGGAAAGGCCTGGAGTGGATGGGAATCATCTATCCTTATCACTCTGATACCCAATACACCCCGTCCTTCCAAGGCCAGGTCACCATCTCAGCCGACGAGTCCACCACCACCCCCTACCTGCACTTGAGCAGCCTGAAGGCCTCGGACACCGCCATATATTACTGTGTGAGACTCAGGGGAGATTTTGTCCCAGTGGTGTTCGCCCCCTACTTTGACTCCTGGGGCCAGGGAACTCTGGTCACC |
| D74H//S80T//W102L//M112I//G121D//R124P//V140L | | G204A//G220C//G239C//G305T//G336A//G362A//G371C//G418C | ATGGGATGGTCATGTATCATCCTTTTTCTAGTAGCAACTGCAACCGGTGTACATTCTGAGGTGCAGCTGGTGGAGTCTGGAGCAGAGGTGAAAAAGCCGGGGGAGTCTCTGAAGATCTCCTGTAAGGCCTCTGGATACAGGTTCACCAACTACTGGATCGGCTGGGTGCGCCAGATGCCCGGGAAAGGCCTGGAGTGGATGGGAATCATCTATCCTTATCACTCTGATACCCAATACACCCCGTCCTTCCAAGGCCAGGTCACCATCTCAGCCGACGAGTCCACCACCACCGCCTACCTGCACTTGAGCAGCCTGAAGGCCTCGGACACCGCCATATATTACTGTGTGAGACTCAGGGGAGATTTTGTCCCAGTGGTGTTCGCCCCCTACTTTGACTCCTGGGGCCAGGGAACTCTGCTCACC |
| D74H//W102L//M112I//G121D//R124P | | G220C//G305T//G336A//G362A//G371C | ATGGGATGGTCATGTATCATCCTTTTTCTAGTAGCAACTGCAACCGGTGTACATTCTGAGGTGCAGCTGGTGGAGTCTGGAGCAGAGGTGAAAAAGCCGGGGGAGTCTCTGAAGATCTCCTGTAAGGCCTCTGGATACAGGTTCACCAACTACTGGATCGGCTGGGTGCGCCAGATGCCCGGGAAAGGCCTGGAGTGGATGGGGATCATCTATCCTTATCACTCTGATACCCAATACAGCCCGTCCTTCCAAGGCCAGGTCACCATCTCAGCCGACGAGTCCACCACCACCGCCTACCTGCACTTGAGCAGCCTGAAGGCCTCGGACACCGCCATATATTACTGTGTGAGACTCAGGGGAGATTTTGTCCCAGTGGTGTTCGCCCCCTACTTTGACTCCTGGGGCCAGGGAACTCTGGTCACC |
| G121D//R124P |  | G362A//G371C | ATGGGATGGTCATGTATCATCCTTTTTCTAGTAGCAACTGCAACCGGTGTACATTCTGAGGTGCAGCTGGTGGAGTCTGGAGCAGAGGTGAAAAAGCCGGGGGAGTCTCTGAAGATCTCCTGTAAGGCCTCTGGATACAGGTTCACCAACTACTGGATCGGCTGGGTGCGCCAGATGCCCGGGAAAGGCCTGGAGTGGATGGGGATCATCTATCCTTATGACTCTGATACCCAATACAGCCCGTCCTTCCAAGGCCAGGTCACCATCTCAGCCGACGAGTCCACCACCACCGCCTACCTGCACTGGAGCAGCCTGAAGGCCTCGGACACCGCCATGTATTACTGTGTGAGACTCAGGGGAGATTTTGTCCCAGTGGTGTTCGCCCCCTACTTTGACTCCTGGGGCCAGGGAACTCTGGTCACC |
| D74H//S80T//W102L//S104T//M112I//G121D//R124P | | G204A//G220C//G239C//G305T//G311C//G336A//G362A//G371C | ATGGGATGGTCATGTATCATCCTTTTTCTAGTAGCAACTGCAACCGGTGTACATTCTGAGGTGCAGCTGGTGGAGTCTGGAGCAGAGGTGAAAAAGCCGGGGGAGTCTCTGAAGATCTCCTGTAAGGCCTCTGGATACAGGTTCACCAACTACTGGATCGGCTGGGTGCGCCAGATGCCCGGGAAAGGCCTGGAGTGGATGGGAATCATCTATCCTTATCACTCTGATACCCAATACACCCCGTCCTTCCAAGGCCAGGTCACCATCTCAGCCGACGAGTCCACCACCACCGCCTACCTGCACTTGAGCACCCTGAAGGCCTCGGACACCGCCATATATTACTGTGTGAGACTCAGGGGAGATTTTGTCCCAGTGGTGTTCGCCCCCTACTTTGACTCCTGGGGCCAGGGAACTCTGGTCACC |
| D74H//S80T//W102L//G121E | | G204A//G220C//G239C//G305T//G362A//T363A | ATGGGATGGTCATGTATCATCCTTTTTCTAGTAGCAACTGCAACCGGTGTACATTCTGAGGTGCAGCTGGTGGAGTCTGGAGCAGAGGTGAAAAAGCCGGGGGAGTCTCTGAAGATCTCCTGTAAGGCCTCTGGATACAGGTTCACCAACTACTGGATCGGCTGGGTGCGCCAGATGCCCGGGAAAGGCCTGGAGTGGATGGGAATCATCTATCCTTATCACTCTGATACCCAATACACCCCGTCCTTCCAAGGCCAGGTCACCATCTCAGCCGACGAGTCCACCACCACCGCCTACCTGCACTTGAGCAGCCTGAAGGCCTCGGACACCGCCATGTATTACTGTGTGAGACTCAGGGGAGAATTTGTCCGAGTGGTGTTCGCCCCCTACTTTGACTCCTGGGGCCAGGGAACTCTGGTCACC |
| W102L//M112I//G121D//R124P | | G305T//G336A//G362A//G371C | ATGGGATGGTCATGTATCATCCTTTTTCTAGTAGCAACTGCAACCGGTGTACATTCTGAGGTGCAGCTGGTGGAGTCTGGAGCAGAGGTGAAAAAGCCGGGGGAGTCTCTGAAGATCTCCTGTAAGGCCTCTGGATACAGGTTCACCAACTACTGGATCGGCTGGGTGCGCCAGATGCCCGGGAAAGGCCTGGAGTGGATGGGGATCATCTATCCTTATGACTCTGATACCCAATACAGCCCGTCCTTCCAAGGCCAGGTCACCATCTCAGCCGACGAGTCCACCACCACCGCCTACCTGCACTTGAGCAGCCTGAAGGCCTCGGACACCGCCATATATTACTGTGTGAGACTCAGGGGAGATTTTGTCCCAGTGGTGTTCGCCCCCTACTTTGACTCCTGGGGCCAGGGAACTCTGGTCACC |
| D74H//S80T//W102L//L105R//M112I//G121D//R124P | | G204A//G220C//G239C//G305T//T314G//G336A//G362A//G371C | ATGGGATGGTCATGTATCATCCTTTTTCTAGTAGCAACTGCAACCGGTGTACATTCTGAGGTGCAGCTGGTGGAGTCTGGAGCAGAGGTGAAAAAGCCGGGGGAGTCTCTGAAGATCTCCTGTAAGGCCTCTGGATACAGGTTCACCAACTACTGGATCGGCTGGGTGCGCCAGATGCCCGGGAAAGGCCTGGAGTGGATGGGAATCATCTATCCTTATCACTCTGATACCCAATACACCCCGTCCTTCCAAGGCCAGGTCACCATCTCAGCCGACGAGTCCACCACCACCGCCTACCTGCACTTGAGCAGCCGGAAGGCCTCGGACACCGCCATATATTACTGTGTGAGACTCAGGGGAGATTTTGTCCCAGTGGTGTTCGCCCCCTACTTTGACTCCTGGGGCCAGGGAACTCTGGTCACC |
| W52C//G121E |  | G156C//G362A//T363A | ATGGGATGGTCATGTATCATCCTTTTTCTAGTAGCAACTGCAACCGGTGTACATTCTGAGGTGCAGCTGGTGGAGTCTGGAGCAGAGGTGAAAAAGCCGGGGGAGTCTCTGAAGATCTCCTGTAAGGCCTCTGGATACAGGTTCACCAACTACTGCATCGGCTGGGTGCGCCAGATGCCCGGGAAAGGCCTGGAGTGGATGGGGATCATCTATCCTTATGACTCTGATACCCAATACAGCCCGTCCTTCCAAGGCCAGGTCACCATCTCAGCCGACGAGTCCACCACCACCGCCTACCTGCACTGGAGCAGCCTGAAGGCCTCGGACACCGCCATGTATTACTGTGTGAGACTCAGGGGAGAATTTGTCCGAGTGGTGTTCGCCCCCTACTTTGACTCCTGGGGCCAGGGAACTCTGGTCACC |
| G121E//V140L |  | G362A//T363A//G418C | ATGGGATGGTCATGTATCATCCTTTTTCTAGTAGCAACTGCAACCGGTGTACATTCTGAGGTGCAGCTGGTGGAGTCTGGAGCAGAGGTGAAAAAGCCGGGGGAGTCTCTGAAGATCTCCTGTAAGGCCTCTGGATACAGGTTCACCAACTACTGGATCGGCTGGGTGCGCCAGATGCCCGGGAAAGGCCTGGAGTGGATGGGGATCATCTATCCTTATGACTCTGATACCCAATACAGCCCGTCCTTCCAAGGCCAGGTCACCATCTCAGCCGACGAGTCCACCACCACCGCCTACCTGCACTGGAGCAGCCTGAAGGCCTCGGACACCGCCATGTATTACTGTGTGAGACTCAGGGGAGAATTTGTCCGAGTGGTGTTCGCCCCCTACTTTGACTCCTGGGGCCAGGGAACTCTGCTCACC |
| R47S//R57H//G121E |  | G141C//G170A//G362A//T363A | ATGGGATGGTCATGTATCATCCTTTTTCTAGTAGCAACTGCAACCGGTGTACATTCTGAGGTGCAGCTGGTGGAGTCTGGAGCAGAGGTGAAAAAGCCGGGGGAGTCTCTGAAGATCTCCTGTAAGGCCTCTGGATACAGCTTCACCAACTACTGGATCGGCTGGGTGCACCAGATGCCCGGGAAAGGCCTGGAGTGGATGGGGATCATCTATCCTTATGACTCTGATACCCAATACAGCCCGTCCTTCCAAGGCCAGGTCACCATCTCAGCCGACGAGTCCACCACCACCGCCTACCTGCACTGGAGCAGCCTGAAGGCCTCGGACACCGCCATGTATTACTGTGTGAGACTCAGGGGAGAATTTGTCCGAGTGGTGTTCGCCCCCTACTTTGACTCCTGGGGCCAGGGAACTCTGGTCACC |
| W102L//G121E |  | G305T//G362A//T363A | ATGGGATGGTCATGTATCATCCTTTTTCTAGTAGCAACTGCAACCGGTGTACATTCTGAGGTGCAGCTGGTGGAGTCTGGAGCAGAGGTGAAAAAGCCGGGGGAGTCTCTGAAGATCTCCTGTAAGGCCTCTGGATACAGGTTCACCAACTACTGGATCGGCTGGGTGCGCCAGATGCCCGGGAAAGGCCTGGAGTGGATGGGGATCATCTATCCTTATGACTCTGATACCCAATACAGCCCGTCCTTCCAAGGCCAGGTCACCATCTCAGCCGACGAGTCCACCACCACCGCCTACCTGCACTTGAGCAGCCTGAAGGCCTCGGACACCGCCATGTATTACTGTGTGAGACTCAGGGGAGAATTTGTCCGAGTGGTGTTCGCCCCCTACTTTGACTCCTGGGGCCAGGGAACTCTGGTCACC |
| WT |  | WT | ATGGGATGGTCATGTATCATCCTTTTTCTAGTAGCAACTGCAACCGGTGTACATTCTGAGGTGCAGCTGGTGGAGTCTGGAGCAGAGGTGAAAAAGCCGGGGGAGTCTCTGAAGATCTCCTGTAAGGCCTCTGGATACAGGTTCACCAACTACTGGATCGGCTGGGTGCGCCAGATGCCCGGGAAAGGCCTGGAGTGGATGGGGATCATCTATCCTTATGACTCTGATACCCAATACAGCCCGTCCTTCCAAGGCCAGGTCACCATCTCAGCCGACGAGTCCACCACCACCGCCTACCTGCACTGGAGCAGCCTGAAGGCCTCGGACACCGCCATGTATTACTGTGTGAGACTCAGGGGAGGTTTTGTCCGAGTGGTGTTCGCCCCCTACTTTGACTCCTGGGGCCAGGGAACTCTGGTCACC |
| M112I//G121D//R124P |  | G336A//G362A//G371C | ATGGGATGGTCATGTATCATCCTTTTTCTAGTAGCAACTGCAACCGGTGTACATTCTGAGGTGCAGCTGGTGGAGTCTGGAGCAGAGGTGAAAAAGCCGGGGGAGTCTCTGAAGATCTCCTGTAAGGCCTCTGGATACAGGTTCACCAACTACTGGATCGGCTGGGTGCGCCAGATGCCCGGGAAAGGCCTGGAGTGGATGGGGATCATCTATCCTTATGACTCTGATACCCAATACAGCCCGTCCTTCCAAGGCCAGGTCACCATCTCAGCCGACGAGTCCACCACCACCGCCTACCTGCACTGGAGCAGCCTGAAGGCCTCGGACACCGCCATATATTACTGTGTGAGACTCAGGGGAGATTTTGTCCCAGTGGTGTTCGCCCCCTACTTTGACTCCTGGGGCCAGGGAACTCTGGTCACC |
| W102L//M112I//G121E |  | G305T//G336A//G362A//T363A | ATGGGATGGTCATGTATCATCCTTTTTCTAGTAGCAACTGCAACCGGTGTACATTCTGAGGTGCAGCTGGTGGAGTCTGGAGCAGAGGTGAAAAAGCCGGGGGAGTCTCTGAAGATCTCCTGTAAGGCCTCTGGATACAGGTTCACCAACTACTGGATCGGCTGGGTGCGCCAGATGCCCGGGAAAGGCCTGGAGTGGATGGGGATCATCTATCCTTATGACTCTGATACCCAATACAGCCCGTCCTTCCAAGGCCAGGTCACCATCTCAGCCGACGAGTCCACCACCACCGCCTACCTGCACTTGAGCAGCCTGAAGGCCTCGGACACCGCCATATATTACTGTGTGAGACTCAGGGGAGAATTTGTCCGAGTGGTGTTCGCCCCCTACTTTGACTCCTGGGGCCAGGGAACTCTGGTCACC |
| H101Q//G121E |  | C303G//G362A//T363A | ATGGGATGGTCATGTATCATCCTTTTTCTAGTAGCAACTGCAACCGGTGTACATTCTGAGGTGCAGCTGGTGGAGTCTGGAGCAGAGGTGAAAAAGCCGGGGGAGTCTCTGAAGATCTCCTGTAAGGCCTCTGGATACAGGTTCACCAACTACTGGATCGGCTGGGTGCGCCAGATGCCCGGGAAAGGCCTGGAGTGGATGGGGATCATCTATCCTTATGACTCTGATACCCAATACAGCCCGTCCTTCCAAGGCCAGGTCACCATCTCAGCCGACGAGTCCACCACCACCGCCTACCTGCAGTGGAGCAGCCTGAAGGCCTCGGACACCGCCATGTATTACTGTGTGAGACTCAGGGGAGAATTTGTCCGAGTGGTGTTCGCCCCCTACTTTGACTCCTGGGGCCAGGGAACTCTGGTCACC |
| I39M//H101Q//G121E |  | C117G//C303G//G362A//T363A | ATGGGATGGTCATGTATCATCCTTTTTCTAGTAGCAACTGCAACCGGTGTACATTCTGAGGTGCAGCTGGTGGAGTCTGGAGCAGAGGTGAAAAAGCCGGGGGAGTCTCTGAAGATGTCCTGTAAGGCCTCTGGATACAGGTTCACCAACTACTGGATCGGCTGGGTGCGCCAGATGCCCGGGAAAGGCCTGGAGTGGATGGGGATCATCTATCCTTATGACTCTGATACCCAATACAGCCCGTCCTTCCAAGGCCAGGTCACCATCTCAGCCGACGAGTCCACCACCACCGCCTACCTGCAGTGGAGCAGCCTGAAGGCCTCGGACACCGCCATGTATTACTGTGTGAGACTCAGGGGAGAATTTGTCCGAGTGGTGTTCGCCCCCTACTTTGACTCCTGGGGCCAGGGAACTCTGGTCACC |
| P60S//G121E |  | C178T//G362A//T363A | ATGGGATGGTCATGTATCATCCTTTTTCTAGTAGCAACTGCAACCGGTGTACATTCTGAGGTGCAGCTGGTGGAGTCTGGAGCAGAGGTGAAAAAGCCGGGGGAGTCTCTGAAGATCTCCTGTAAGGCCTCTGGATACAGGTTCACCAACTACTGGATCGGCTGGGTGCGCCAGATGTCCGGGAAAGGCCTGGAGTGGATGGGGATCATCTATCCTTATGACTCTGATACCCAATACAGCCCGTCCTTCCAAGGCCAGGTCACCATCTCAGCCGACGAGTCCACCACCACCGCCTACCTGCACTGGAGCAGCCTGAAGGCCTCGGACACCGCCATGTATTACTGTGTGAGACTCAGGGGAGAATTTGTCCGAGTGGTGTTCGCCCCCTACTTTGACTCCTGGGGCCAGGGAACTCTGGTCACC |
| C41Y//G121E |  | G122A//G362A//T363A | ATGGGATGGTCATGTATCATCCTTTTTCTAGTAGCAACTGCAACCGGTGTACATTCTGAGGTGCAGCTGGTGGAGTCTGGAGCAGAGGTGAAAAAGCCGGGGGAGTCTCTGAAGATCTCCTATAAGGCCTCTGGATACAGGTTCACCAACTACTGGATCGGCTGGGTGCGCCAGATGCCCGGGAAAGGCCTGGAGTGGATGGGGATCATCTATCCTTATGACTCTGATACCCAATACAGCCCGTCCTTCCAAGGCCAGGTCACCATCTCAGCCGACGAGTCCACCACCACCGCCTACCTGCACTGGAGCAGCCTGAAGGCCTCGGACACCGCCATGTATTACTGTGTGAGACTCAGGGGAGAATTTGTCCGAGTGGTGTTCGCCCCCTACTTTGACTCCTGGGGCCAGGGAACTCTGGTCACC |
| I39M//G121E |  | C117G//G362A//T363A | ATGGGATGGTCATGTATCATCCTTTTTCTAGTAGCAACTGCAACCGGTGTACATTCTGAGGTGCAGCTGGTGGAGTCTGGAGCAGAGGTGAAAAAGCCGGGGGAGTCTCTGAAGATGTCCTGTAAGGCCTCTGGATACAGGTTCACCAACTACTGGATCGGCTGGGTGCGCCAGATGCCCGGGAAAGGCCTGGAGTGGATGGGGATCATCTATCCTTATGACTCTGATACCCAATACAGCCCGTCCTTCCAAGGCCAGGTCACCATCTCAGCCGACGAGTCCACCACCACCGCCTACCTGCACTGGAGCAGCCTGAAGGCCTCGGACACCGCCATGTATTACTGTGTGAGACTCAGGGGAGAATTTGTCCGAGTGGTGTTCGCCCCCTACTTTGACTCCTGGGGCCAGGGAACTCTGGTCACC |
| W102C//G121E |  | G306T//G362A//T363A | ATGGGATGGTCATGTATCATCCTTTTTCTAGTAGCAACTGCAACCGGTGTACATTCTGAGGTGCAGCTGGTGGAGTCTGGAGCAGAGGTGAAAAAGCCGGGGGAGTCTCTGAAGATCTCCTGTAAGGCCTCTGGATACAGGTTCACCAACTACTGGATCGGCTGGGTGCGCCAGATGCCCGGGAAAGGCCTGGAGTGGATGGGGATCATCTATCCTTATGACTCTGATACCCAATACAGCCCGTCCTTCCAAGGCCAGGTCACCATCTCAGCCGACGAGTCCACCACCACCGCCTACCTGCACTGTAGCAGCCTGAAGGCCTCGGACACCGCCATGTATTACTGTGTGAGACTCAGGGGAGAATTTGTCCGAGTGGTGTTCGCCCCCTACTTTGACTCCTGGGGCCAGGGAACTCTGGTCACC |
|  |  |  |  |

Table S3 : gRNA sequences binding to the Ig gene sense (s) or antisense (as) strands

| Name | Sequence |
| --- | --- |
| gRNA 98s 24 | AGCAGAGGTGAAAAAGCCGG*GGG* |
| gRNA 193s 119 | TGCCCGGGAAAGGCCTGGAG*TGG* |
| gRNA 314s 240 | CCTGCACTGGAGCAGCCTGA*AGG* |
| gRNA 357s 283 | TACTGTGTGAGACTCAGGGG*AGG* |
| gRNA 131as 71 | GTTGGTGAACCTGTATCCAG*AGG* |
| gRNA 184as124 | ATCCACTCCAGGCCTTTCCC*GGG* |
| gRNA 293as 233 | GCTCCAGTGCAGGTAGGCGG*TGG* |
| gRNA 296as 236 | GCTGCTCCAGTGCAGGTAGG*CGG* |
| gRNA 428as 368 | CTTGGTCGACGCTGAGGAGA*CGG* |

PAM sequences are underlined
